# Digital Health Interventions for Depression and Anxiety Among People With Chronic Conditions: Scoping Review

**DOI:** 10.2196/38030

**Published:** 2022-09-26

**Authors:** Amika Shah, Neesha Hussain-Shamsy, Gillian Strudwick, Sanjeev Sockalingam, Robert P Nolan, Emily Seto

**Affiliations:** 1 Institute of Health Policy, Management and Evaluation Dalla Lana School of Public Health University of Toronto Toronto, ON Canada; 2 Centre for Global eHealth Innovation University Health Network Toronto, ON Canada; 3 Centre for Addiction and Mental Health Toronto, ON Canada; 4 University Health Network Toronto, ON Canada; 5 Department of Psychiatry University of Toronto Toronto, ON Canada; 6 Cardiac eHealth Toronto General Hospital University Health Network Toronto, ON Canada; 7 Institute of Medical Science University of Toronto Toronto, ON Canada

**Keywords:** depression, anxiety, multiple chronic conditions, chronic disease, mental health, psychiatry, digital health, eHealth, telehealth, mobile health, mHealth, telemedicine

## Abstract

**Background:**

Chronic conditions are characterized by their long duration (≥1 year), need for ongoing medical attention, and limitations in activities of daily living. These can often co-occur with depression and anxiety as common and detrimental comorbidities among the growing population living with chronic conditions. Digital health interventions (DHIs) hold promise in overcoming barriers to accessing mental health support for these individuals; however, the design and implementation of DHIs for depression and anxiety in people with chronic conditions are yet to be explored.

**Objective:**

This study aimed to explore what is known in the literature regarding DHIs for the prevention, detection, or treatment of depression and anxiety among people with chronic conditions.

**Methods:**

A scoping review of the literature was conducted using the Arksey and O’Malley framework. Searches of the literature published in 5 databases between 1990 and 2019 were conducted in April 2019 and updated in March 2021. To be included, studies must have described a DHI tested with, or designed for, the prevention, detection, or treatment of depression or anxiety in people with common chronic conditions (arthritis, asthma, diabetes mellitus, heart disease, chronic obstructive pulmonary disease, cancer, stroke, and Alzheimer disease or dementia). Studies were independently screened by 2 reviewers against the inclusion and exclusion criteria. Both quantitative and qualitative data were extracted, charted, and synthesized to provide a descriptive summary of the trends and considerations for future research.

**Results:**

Database searches yielded 11,422 articles across the initial and updated searches, 53 (0.46%) of which were included in this review. DHIs predominantly sought to provide treatment (44/53, 83%), followed by detection (5/53, 9%) and prevention (4/53, 8%). Most DHIs were focused on depression (36/53, 68%), guided (32/53, 60%), tailored to chronic physical conditions (19/53, 36%), and delivered through web-based platforms (20/53, 38%). Only 2 studies described the implementation of a DHI.

**Conclusions:**

As a growing research area, DHIs offer the potential to address the gap in care for depression and anxiety among people with chronic conditions; however, their implementation in standard care is scarce. Although stepped care has been identified as a promising model to implement efficacious DHIs, few studies have investigated the use of DHIs for depression and anxiety among chronic conditions using such models. In developing stepped care, we outlined DHI tailoring, guidance, and intensity as key considerations that require further research.

## Introduction

### Background

Chronic conditions often co-occur despite an emphasis on their singular occurrence in health interventions, research, and care [1,2]. Chronic conditions persist for long durations (≥1 year), require ongoing medical attention, and limit activities of daily living [3]. In Canada, national data have identified several common chronic conditions, including arthritis, asthma, diabetes mellitus, heart disease, chronic obstructive pulmonary disease, cancer, stroke, mood and anxiety disorders, and Alzheimer disease and related dementias [4]. When poorly managed, these conditions have been associated with negative outcomes, such as poor quality of life [5], increased health care use [6], and higher costs [7].

Concomitant mood and anxiety disorders, such as depression and anxiety, are of particular concern because of their high prevalence [8-10] and burden [11-13] but are often underdetected and undertreated [14,15]. Prevalence estimates of depression (9.3%-23% [16]) and anxiety (2.9%-8.8% [12]) range widely and often vary based on the type and severity of co-occurring chronic conditions. Nevertheless, several studies have suggested that these rates are higher among patients with co-occurring chronic conditions than among those without the conditions [17,18]. Independently, depression has been associated with decrements in physical health outcomes and quality of life [16] for people with chronic conditions, including poor chronic disease self-management [19], worse outcomes for co-occurring physical conditions [20], adverse health system outcomes such as higher use of urgent care [21], and higher costs [22]. Similar impacts have been found for anxiety with chronic conditions [19,23], although this has been less studied. People with chronic conditions face additional challenges in accessing health support in the face of multiple appointments, transportation barriers, and treatment burdens [24]. These barriers may be especially pronounced for those with depression or anxiety, who may have added challenges in accessing support owing to their mental health status and stigma [25].

Digital health interventions (DHIs), that is, health interventions delivered through digital technologies, may help overcome barriers to both delivering mental health care (ie, stigma and access) and care for chronic conditions (ie, communication barriers and lack of appropriate information) [26,27] because of their ability to be accessed remotely, discreetly, and in real time. Existing meta-analytic research [28-34] suggests that DHIs are effective interventions for improving both psychological and disease-specific outcomes for populations with chronic conditions, with small to moderate effect sizes [28,30,32]. The most recent and largest of these meta-analyses reported that self-guided web-based interventions were associated with significant reductions in depression and anxiety compared with usual care or waitlist control, with small effect sizes [34]. However, existing reviews have been limited to a narrow set of therapeutic strategies (eg, self-help [34,35], mindfulness interventions [31,36], cognitive behavioral therapy [CBT]; [28,30,32]), technologies (eg, web-based platforms) [28,30,32,34-36], and outcomes (eg, effectiveness) [28,30-37], leaving the design and implementation of such technologies less clear.

### Objectives

To complement previous reviews, this study sought to examine a wider range of technologies (mobile apps, telemonitoring systems, etc), functions DHIs may serve with respect to depression and anxiety (prevention, detection, or treatment), and study designs (qualitative, quantitative, pilot studies, etc). Specifically, this scoping review aimed to explore what is known about DHIs to prevent, detect, and treat depression or anxiety among people living with chronic conditions. To our knowledge, this is the first scoping review of primary research on DHIs for depression or anxiety in people with chronic conditions.

## Methods

### Overview

This scoping review was based on the following framework for conducting scoping reviews as developed by Arksey and O’Malley [38] and refined by Levac et al [39]: (1) identifying the research questions in light of the research purpose; (2) identifying relevant studies while balancing feasibility with comprehensiveness; (3) identifying the study selection criteria using an iterative team approach; (4) charting the data to provide both a numerical summary and thematic analysis; and (5) reporting the results with implications for policy, practice, and research identified [1]. In addition to the framework by Arksey and O’Malley [38] and Levac et al [39], this review adhered to the scoping review guidelines outlined by the JBI [40]. Informed by these frameworks, the actions taken at each stage of the scoping review process are described in the following sections [2].

### Stage 1: Identifying the Research Question

This scoping review was guided by the following research question: What is known about the use of DHIs to support the prevention, detection, or treatment of depression or anxiety among people with chronic conditions? To address this research question, this study sought to (1) describe the nature and extent of DHIs to support the prevention, detection, and treatment of depression or anxiety among people with chronic conditions; (2) describe existing research and its overall findings; and (3) identify gaps and opportunities for future research.

### Stage 2: Identifying Relevant Studies

This scoping review was designed and reported in line with the PRISMA-ScR (Preferred Reporting Items for Systematic Reviews and Meta-Analyses extension for Scoping Reviews) checklist (Multimedia Appendix 1) [41]. A protocol was developed to guide this review; however, it was not registered. Studies relevant to the research question were identified through searches of the following databases: Embase, CINAHL, PsycINFO, MEDLINE, and the Cochrane Library using terms related to the concepts of “digital health,” “chronic disease,” “depression,” and “anxiety.” As recommended by the Joanna Briggs Institute [40], a 3-step process was used to develop the search strategy. First, initial searches of MEDLINE and PsycINFO were conducted to identify the terms used in the titles and abstracts of the articles. This first step helped identify relevant keywords and subject headings. Second, terms identified in the initial searches were arranged into a search strategy for MEDLINE, which was later tailored and revised for other databases. An iterative team approach was used to develop the search strategy [39] by consulting with a discipline-specific research librarian and seeking feedback from the research team. After tailoring the initial search strategy for MEDLINE to the rest of the databases (Embase, CINAHL, PsycINFO, and the Cochrane Library), searches of all databases were conducted initially in April 2019 and later updated in March 2021 to capture new publications between April 2019 and March 2021. The search strategy for all databases was saved to ensure reproducibility of the search results (see Multimedia Appendix 2 for the search strategy for MEDLINE). In the third step, the reference lists of relevant studies were examined in other studies that could be pertinent to the research question. Journals related to digital health (*Journal of Medical Internet Research* and *Internet Interventions*) were also hand searched for potentially relevant articles.

### Stage 3: Study Selection

The results of the research strategy were saved in the reference management software Mendeley (Elsevier) to identify duplicates and were then exported to Rayyan [42], an internet-based platform designed to expedite the process of screening articles in systematic reviews. As outlined by Arksey and O’Malley [38], studies were selected based on their relevance to the scoping review question rather than their methodological rigor. The studies were reviewed based on the inclusion and exclusion criteria listed in Textbox 1.

**Study eligibility criteria.**

**Inclusion criteria**

- Article published in English in a peer-reviewed academic journal
- Published after 1990, owing to our interest in newer technologies
- Describe a digital health intervention (DHI) defined as a health intervention delivered via digital technologies including but not limited to web-based platforms, videoconferencing, mobile phone apps, SMS text messages, email, wearable devices, and monitoring sensors
- Study population of individuals aged ≥18 years with one or more of the common chronic conditions identified by the Public Health Agency of Canada (PHAC) [43]: arthritis, asthma, diabetes mellitus, heart disease, chronic obstructive pulmonary disease, cancer, stroke, and Alzheimer disease and related dementias [4]. Although depression and anxiety are also deemed common chronic conditions per PHAC, these conditions were selected because of their high rate of comorbidity with the other common chronic conditions listed [8-10].
- Intervention objective, in whole or in part, related to the prevention, detection, or treatment of depression or anxiety. This was operationalized by studies that either
  - Included a study population with depression or anxiety in addition to one or more of the common chronic conditions outlined by PHAC [43].
  - Explicitly stated that the intervention sought to prevent, detect, or treat depression or anxiety among those with one or more of the common chronic conditions outlined by PHAC [43].

**Exclusion criteria**

- Editorials, case reports, abstracts, posters, or dissertations
- Interventions that solely used the phone calling functionality of telephones [44] (including interactive voice response) were not included in our definition of DHIs stated previously
- Interventions for other mood or mental disorders beyond depression and anxiety
- Studies with a mixed youth and young adult population (eg, [45])
- Lifestyle or survivorship programs

Overall, 2 reviewers (AS and NHS) screened studies yielded by the search strategy by title and abstract and excluded studies if they met any of the exclusion criteria. Rayyan QCRI [42] was used to coordinate screening between the reviewers. Efforts were made to retain studies until full-text review if the reviewers were unsure about the eligibility of the studies with respect to the scoping review criteria. Review articles (ie, systematic reviews, meta-analyses, meta-syntheses, scoping reviews, narrative reviews, rapid reviews, critical reviews, and integrative reviews) were collected, and the reference lists were scanned for potentially relevant articles. Once the reference lists were scanned and potentially relevant articles were identified, the review articles were excluded. The full text of the remaining studies was reviewed according to the inclusion and exclusion criteria. Studies that met the inclusion criteria and those that did not meet the exclusion criteria were included in the scoping review. Studies that met the exclusion criteria were excluded, and the reasons were recorded. Disagreements between the reviewers were resolved through discussion until a consensus was reached, and a third reviewer (ES) was engaged when needed to resolve any remaining conflicts.

### Stage 4: Data Collection

Two reviewers (AS and NHS) independently extracted data from the studies included in the scoping review using a data extraction form. A preliminary data extraction form was drafted in Microsoft Excel with the following column headings: title, year of publication, study details (eg, location, objective, research design, methods, eligibility criteria, target population, conditions, outcomes measured, and findings), and intervention details (eg, name, goal, technology, therapeutic components, and guidance [where any in-person or digital correspondence with a human was considered guided]). The form was modified and revised as necessary during the charting process. Discrepancies between the reviewers regarding the extracted data were resolved through discussion.

### Stage 5: Data Summary and Synthesis of Results

Data analysis was performed using the following steps. First, the results of the nature and extent of the studies and interventions were summarized. Second, details related to the findings of existing studies were collated, and trends were discussed with both reviewers (AS and NHS) to identify opportunities for further research.

## Results

### Overview

**Figure 1:**
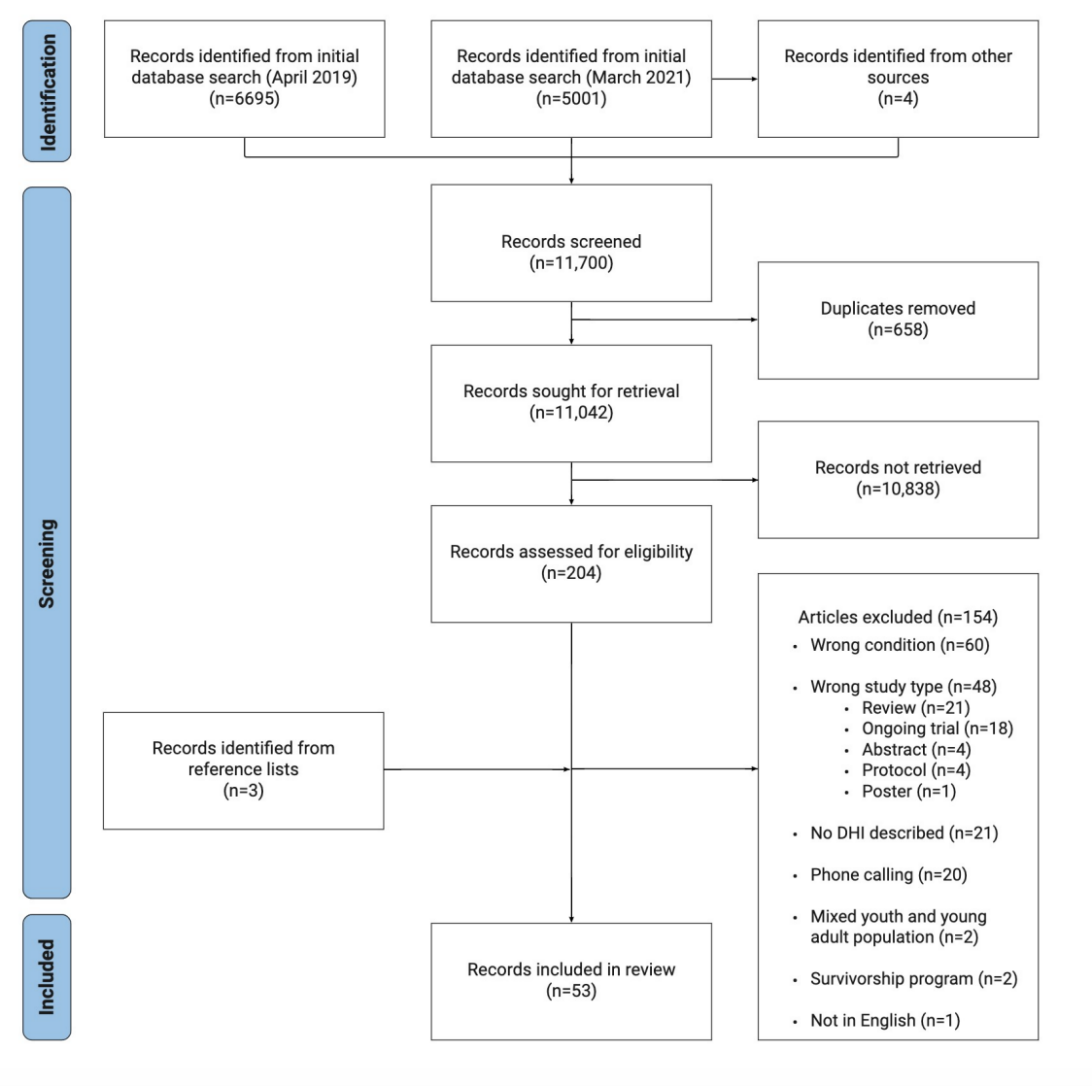
PRISMA (Preferred Reporting Items for Systematic Reviews and Meta-Analyses) flow diagram. DHI: digital health intervention.

Initial searches of 5 databases in April 2019 yielded 6695 results, with one additional article identified through hand searches of a relevant journal (*Journal of Medical Internet Research*; Figure 1). After removing duplicates (n=381), two authors (AS and NHS) screened 6315 records by title and abstract. At this stage, 6228 articles were excluded, including 74 reviews whose reference lists were checked for potentially relevant articles. An additional 3 relevant studies were identified from these reference lists. This left 87 articles to be assessed by full text against the inclusion and exclusion criteria by an author (AS). Of these 87 articles, 61 (70%) were excluded for the following reasons: the study population or intervention goal was not depression or anxiety among individuals with common chronic conditions as identified by the Public Health Agency of Canada [43] (32/61, 52%), the intervention was not a DHI (15/61, 25%), and the intervention relied exclusively on phone calls (14/61, 23%). This resulted in a total of 26 articles. A search update was conducted using the same search strategy in March 2021 for studies published between April 2019 and March 2021. The search update yielded 5001 articles, 4727 (94.52%) and 4610 (92.18%) of which were reviewed in duplicate (AS and NHS) and excluded by title and abstract, respectively. The full text of the remaining 117 studies was reviewed, with 93 (79.5%) studies excluded, resulting in another 24 (20.5%) articles included. An additional 3 articles were identified by screening the reference lists. Overall, 53 articles [46-99] were included in this review (Multimedia Appendix 3 [46,47,50,52,54,55,57-60,62,​^64^-^66^,^70^-^72^,^74^,^77^,^78^,^80^,^81^,^84^,^86^,^88^,^90^-^92^,^95^,^96^,^98^,^99^]). The following sections outline the characteristics of the included studies and report the details related to study objectives 1 and 2.

### Study Characteristics

#### Publication Frequency

Table 1 shows the frequency of the included studies by publication year. From 1990 to 2010, no study met our inclusion criteria, highlighting the recent and emerging nature of this area of research. The first study emerged in 2011, with most studies meeting the criteria published in 2019 (16/53, 30%).

**Table 1:** Number of included articles by year of publication (N=53).

Year	Studies, n (%)
2011	3 (6)
2012	1 (2)
2013	1 (2)
2014	1 (2)
2015	3 (6)
2016	5 (9)
2017	4 (8)
2018	5 (9)
2019	16 (30)
2020	13 (25)
2021	1 (2)

#### Setting

The included studies were predominantly conducted in the United States (15/53, 28%), Sweden (7/53, 13%), Australia (6/53, 11%), Canada (3/53, 6%), and India (4/53, 8%). Other study locations included Germany (3/53, 6%), the Netherlands (2/53, 4%), the United Kingdom (2/53, 4%), China (2/53, 4%), Peru (1/53, 2%), and Jordan (1/53, 2%; Table 2). A total of 4 studies did not specify the study location. On the basis of economy type (as determined by the World Bank classification [100]), studies were largely conducted in high-income countries (38/53, 72%) and to a lesser extent in upper–middle-income countries (4/53, 8%) and lower–middle-income countries (4/53, 8%). There were 3 instances in which studies involved multiple locations; however, these collaborations were between countries with similar economy types: multiple high-income countries (2/53, 4%) and between upper middle-income countries (1/53, 2%).

**Table 2:** Summary of study characteristics (N=53).

		Studies, n (%)	References
**Study location**			
	United States	15 (28)	[48,54,58,62,66,69,76,79,82,84,87,89,94,95,97]
	Sweden	7 (13)	[47,65,77,80,81,86,92]
	Australia	6 (11)	[57,63,85,91,93,96]
	Canada	3 (6)	[67,78,83]
	India	4 (8)	[61,72,73,75]
	Germany	3 (6)	[71,98,99]
	The Netherlands	2 (4)	[52,88]
	United Kingdom	2 (4)	[49,59]
	China	2 (4)	[53,70]
	Peru	1 (2)	[90]
	Jordan	1 (2)	[51]
	Location not reported	4 (8)	[50,55,60,64]
**Economy type**			
	HIC^a^	38 (72)	[47-49,52,54,57-59,62,63,65-67,69,71,76-89,91-99]
	UMIC^b^	4 (8)	[51,53,68,70]
	Lower–middle-income country	4 (8)	[61,72,73,75]
	Multiple HICs	2 (4)	[56,74]
	Multiple UMICs	1 (2)	[68]
**Requirements for study eligibility**			
	Access or ownership of a digital device	13 (25)	[49,59,63,65,73,82,85,87,91,93,95,97,98]
	Internet access	9 (17)	[47,52,59,62,63,65,67,91,98]
	Digital literacy or skills	6 (11)	[48,57,70,74,82,88]
	Owning an email address	3 (6)	[52,74,98]
Sample sex distributions: predominantly female (≥60%)		29 (55)	[49,51,52,54,55,57,60,62-64,68,74,76-81,83,87-91,93,94,97-99]
**Research design**			
	Randomized controlled trial	29 (55)	[49,51-53,57-59,62,65,67,70-72,75,78,79,81,83-85,87-89,91,92,95,96,98,99]
	Quasi-experimental	5 (9)	[47,60,63,68,76]
	Grounded theory	2 (4)	[50,64]
	Observational	1 (2)	[73]
	Case study	1 (2)	[66]
	Phenomenological	1 (2)	[56]
**Methods**			
	Quantitative	23 (43)	[52,58,60,62,65,68,73,74,76,78,83,84,88,89,91,93,99,101-106]
	Qualitative	9 (17)	[50,55,56,64,66,77,80,90,97]
	Multi-methods	8 (15)	[47,48,54,63,69,74,75,89]

^a^HIC: high-income country.

^b^UMIC: upper–middle-income country.

#### Population

The sample size ranged from 6 to 3698. Approximately 40% (22/54) of the included studies specified digital requirements for study eligibility, such as access to or ownership of a digital device (13/53, 25%), internet access (9/53, 17%), digital literacy or skills (6/53, 11%), and owning an email address (3/53, 6%). Most articles reported that the study population was predominantly (≥60%) female (29/53, 55%).

#### Methods

Among the studies that reported a research design, the randomized controlled trial design was typically used (29/53, 55%), followed by quasi-experimental (5/53, 9%), grounded theory (2/53, 4%), observational design (1/53, 2%), case study (1/53, 2%), and phenomenological design (1/53, 2%). Different methods were used among the included studies, including quantitative (23/53, 43%), qualitative (9/53, 17%), and multi-methods research (8/53, 15%). A total of 3 articles [61,85,86] described the development of a DHI or the lessons learned in conducting a study but did not state a qualitative methodology.

### Nature and Extent of DHIs

#### Intervention Purpose and Digital Technologies

The 53 studies included in this review described 36 unique DHIs. The purpose of the interventions was distributed as follows: prevention (4/53, 8%), detection (5/53, 9%), and treatment (44/53, 83%; Table 3). Over the past decade, the technologies most commonly used to deliver these interventions were web-based platforms (20/53, 38%) and mobile devices (17/53, 32%). Telehealth systems (3/53, 6%), electronic health records (2/53, 4%), and virtual reality devices (1/53, 2%) were also used. Several studies have described DHIs that leverage multiple technologies (10/53, 19%).

**Table 3:** Summary of intervention characteristics (N=53).

			Studies, n (%)		References
**Purpose**					
	Prevention	4 (8)		[57,89,93,96]	
	Detection	5 (8)		[56,67,73,76,82]	
	Treatment	44 (83)		[47-55,58-66,68-72,74,75,77-81,83-88,90-92,94,95,97-99]	
**Technology**					
	Web based	20 (38)		[47,49,52,54,58,62,65,74,77,80,81,84-86,88,91-93,96,98]	
	Mobile device	17 (32)		[48,53,56,61,67-70,73,75,79,82,87,89,90,94,97]	
	Telehealth system	3 (6)		[66,71,95]	
	Electronic health records	2 (4)		[72,76]	
	Virtual reality	1 (2)		[51]	
	Multiple technologies	10 (19)		[50,55,57,59,60,63,64,78,83,99]	
**Target conditions (mental)**					
	Depression	36 (68)		[50,52,55-58,60-66,68,69,71-76,78,82-85,87,88,90-93,95,97-99]	
	Anxiety	3 (6)		[51,59,79]	
	Depression and anxiety	14 (26)		[47-49,53,54,67,70,77,80,81,86,89,94,96]	
**Target conditions (physical)**					
	Diabetes	19 (36)		[49,52,61,63,68,72-76,82,85,88,90,91,93,95,98,99]	
	Cancer	13 (25)		[51,53,54,70,77,79-81,86,87,94,96,97]	
	Hypertension	5 (9)		[47,61,68,75,90]	
	Heart failure	3 (6)		[65,71,92]	
	Chronic obstructive pulmonary disease	2 (4)		[56,67]	
	Stroke	1 (2)		[59]	
	Any chronic condition	8 (15)		[50,55,60,62,64,66,69,84]	
	Multiple chronic conditions	6 (11)		[48,57,58,78,83,89]	
**Role of digital health intervention**					
	Sole intervention	42 (79)		[47-52,55,57,59,60,62-65,68,69,71,73,74,77-99]	
	Component of intervention	11 (21)		[53,54,56,58,61,66,67,70,72,75,76]	
**Intervention components**					
	Education	30 (57)		[46,49,50,52,55,59,60,63,64,66,67,69,70,73,74,77,78,80,81,83-86,88-91,93,94,96]	
	Cognitive behavioral therapy	21 (40)		[47,49,51,52,59,63,65,69,70,74,75,77-81,83,85,92-94]	
	Behavioral activation	4 (8)		[46,73,90,91]	
	Problem-solving therapy	2 (4)		[73,91]	
	Acceptance and commitment therapy	1 (2)		[54]	
	Monitoring mental health status or symptoms	14 (26)		[48,49,56,57,63,69,72,78,82,83,85,88,93,98]	
	Peer support	9 (17)		[50,55,60,64,66,77,80,81,86]	
	Communication with health care providers	9 (17)		[48,67,71,72,77,80,81,86,95]	
	Mindfulness	3 (6)		[75,87,97]	
	Chat rooms or forums	7 (13)		[66,77,80,81,84,86,96]	
Tailoring: tailored to chronic physical conditions			19 (36)		[47,48,52,54,59,65,70,73,74,77,79-81,84,86,88,91,92,96]
Guidance: guided			32 (60)		[46,47,50-52,55,58-60,64-66,69-74,77,78,80,81,83,84,86,90-92,94-96,98]
**Guidance provider**					
	Nurse	9 (17)		[47,65,68,70,71,86,90,92,96]	
	Psychologist	6 (11)		[52,57,74,88,91,98]	
	Certified peer specialist	4 (8)		[50,55,60,64]	
	Trained lay individual	2 (4)		[78,83]	
	Allied health professional	2 (4)		[1,2]	
	Study staff members	2 (4)		[58,62]	
	Physician	1 (2)		[59]	
	Multiple professionals	6 (11)		[54,77,80,81,84,99]	
	Unclear	1 (2)		[66]	
**Guidance purpose**					
	Responding to questions	11 (21)		[47,57,62,65,77,80,81,86,92,95,96]	
	Information and feedback	9 (17)		[52,74,80,81,88,95,96,98,99]	
	Promoting engagement and adherence	8 (15)		[50,55,57,60,62,64,91,99]	
	Sending reminders	7 (13)		[47,52,65,74,88,92,98]	
	Offering support	6 (11)		[50,55,60,64,70,91]	
	Monitoring symptoms	4 (8)		[72,78,83,95]	
	Training to use the intervention	4 (8)		[58,68,90,95]	
	Encouragement or positive reinforcement	4 (8)		[57,68,90,95]	
	Moderating forum	3 (6)		[80,81,86]	
	Counseling	2 (4)		[59,72]	
	Check-ins	1 (2)		[54]	
	Unclear	2 (4)		[66,84]	
**Delivery of guidance**					
	Combination	13 (25)		[50,54,55,57,60,64,72,77,90,91,95,98,99]	
	Phone calls	7 (13)		[59,68,71,78,83,84,96]	
	Emails	6 (11)		[47,52,65,74,88,92]	
	Web-based messages	3 (6)		[80,81,86]	
	In-person visits	2 (4)		[58,62]	
	WeChat messages	1 (2)		[70]	
	Unclear	1 (2)		[66]	

#### Target Conditions

Most of the studies included in this review sought to address depression (36/53, 68%) among those with chronic conditions, with some studies (14/53, 26%) focusing on both depression and anxiety. Only 6% (3/32) of studies focused exclusively on anxiety. Regarding co-occurring chronic conditions, most interventions were designed for people with diabetes (19/53, 36%) and cancer (13/53, 25%). Other chronic physical conditions represented in the included studies were hypertension (5/53, 9%), heart failure (3/53, 6%), chronic obstructive pulmonary disease (2/53, 4%), and stroke (1/53, 2%). In addition, 8 studies were for any chronic condition, and 6 studies were for multiple chronic conditions (2 or more chronic physical conditions).

#### Intervention Design and Components

Of the 53 included studies, 42 (79%) described the DHI as the sole intervention, whereas in 11 (21%) studies, the DHI was a component of the intervention. DHIs commonly included education (30/53, 57%), psychological therapy (28/53, 53%; eg, CBT 21/53, 40%; behavioral activation 4/53, 8%; problem-solving therapy 2/53, 4%; and acceptance and commitment therapy 1/53, 2%), and monitoring of mental health status or symptoms (14/53, 26%). However, peer support (9/53, 17%), communication with health care providers (9/53, 17%), mindfulness (3/53, 6%), and chat rooms or forums (7/53, 13%) were also used. Mental health intervention content was frequently tailored to chronic physical conditions (19/53, 36%; Multimedia Appendix 4 [47-99]).

Most interventions included in this review were guided (32/53, 27%), with the frequency at which guidance was provided ranging from as needed to daily (Multimedia Appendix 5 [47-99]). Among the guided interventions, the provider of guidance varied widely across the studies, including nurse specialists, nurse practitioners, or nurse assistants (9/32, 28%); psychologists (6/32, 19%); certified peer supporters (4/32, 13%); trained lay individuals (2/32, 6%); allied health professionals (2/32, 6%; eg, dieticians or social workers 1/32, 3% and pharmacists 1/32, 3%); study staff members (2/32, 6%); and physicians (1/32, 3%). In 19% (6/32) of studies, guidance was provided by multiple professionals (eg, nurses and psychologists, social workers and psychologists, psychology graduate students, and psychologists). In 3% (1/32) of studies, the provider of guidance was unclear. Guidance served a wide range of functions, including responding to questions (11/32, 34%), providing information and feedback (9/32, 28%), promoting engagement and adherence (8/32, 25%), sending reminders (7/32, 22%), offering support (6/32, 19%), monitoring symptoms (4/32, 13%), training to use the intervention (4/32, 13%), providing encouragement or positive reinforcement (4/32, 13%), moderating a forum (3/32, 9%), counseling (2/32, 6%), and check-ins (1/32, 3%). In some studies (2/32, 6%), the purpose of guidance was unclear. Of the studies describing a guided intervention, most (13/32, 41%) used a combination of modalities (eg, in-person, phone calls, and SMS text messages) to provide guidance. In some studies, guidance was offered exclusively through phone calls (7/32, 22%), emails (6/32, 19%), web-based messages (3/32, 9%), in-person visits (2/32, 6%), and WeChat messages (1/32, 3%). In 3% (1/32) of studies, the method of delivery for guidance was unclear.

### Findings of Existing Studies

The objectives of the included studies ranged from design and development (5/53, 9%), feasibility and acceptability (19/53, 36%), determining effectiveness (17/53, 32%) or efficacy (7/53, 13%) of the DHI, and conducting a secondary analysis to explore predictors, mediators, or moderators of DHI outcomes (6/53, 11%; Table 4). Only 4% (2/32) of studies described the implementation of an intervention, one of which [48] described a planned study but was included because of its discussion of the strategies used to implement the DHI. Other study objectives were to describe the adherence and use of a DHI (1/53, 2%), evaluate a prediction model (1/53, 2%), determine the validity of delivering screening tools via text (1/53, 2%), and analyze SMS text messages from a DHI (1/53, 2%). Several studies reported objectives spanning multiple research stages (8/53, 15%). Among the studies investigating the effectiveness (17/53, 32%) or efficacy (7/53, 13%) of a DHI, 54% (13/32) of studies reported significant changes in mental health outcomes. A study reported significant changes in patients’ health behavior (medication adherence [95]) and another in health care provider behavior (depression screening [76]). Multimedia Appendix 3 provides a detailed summary of the findings of each study included in the review.

**Table 4:** Summary of study findings (N=53).

		Studies, n (%)	References
**Study objective**			
	Design and development	5 (9)	[47,61,69,74,86]
	Feasibility and acceptability	19 (36)	[47,50,54,56,59,60,62-64,66,68,70,76,77,80,85,87,90,97]
	Effectiveness	17 (32)	[51-53,58,60,63,65,68,70,72,75,76,78,81,89,93,96]
	Efficacy	7 (13)	[49,57,79,84,91,95,99]
	Secondary analysis	6 (11)	[71,83,88,92,94,98]
	Implementation	2 (4)	[48,70]
	Other	3 (6)	[1-3]
Impact: significant differences in mental health outcomes		13 (25)	[51,52,57,58,62,63,68,70,72,78,81,91,99]

## Discussion

### Principal Findings

This review aimed to explore what is known about DHIs to prevent, detect, and treat depression or anxiety among people with chronic conditions. This review extends the existing meta-analytic evidence of DHIs by reviewing technologies beyond web-based platforms and exploring design and implementation considerations. The findings of this review highlight the significant potential of DHIs to have a profound public health impact on people living with chronic conditions. Per study objective 3, the following section outlines opportunities for further research for this rapidly growing area of investigation: tailoring, guidance, intensity, and stepped care.

### Opportunities for Further Research

#### Tailoring

There was mixed evidence among the studies reviewed regarding the value of tailoring the intervention content to chronic physical conditions. The benefits of both condition-specific and generic DHIs were discussed among the studies reviewed. For instance, van Bastelaar et al [74] found that 80% of the patients reported that a diabetes-specific approach for coping with depression intervention was needed. In a previous review, van Beugan et al [30] highlighted the importance of disease-specific tailoring in attributing the larger improvements in disease-related outcomes observed among these interventions for this feature. However, studies in our review also indicated that generic DHIs were beneficial in addressing depression among people with chronic conditions [58,62,91]. It is possible that certain conditions may require a condition-specific approach owing to the existence of condition-specific mental health constructs (eg, diabetes distress) [93]. However, the growing prevalence of multiple chronic conditions globally and emerging literature on transdiagnostic interventions for depression and anxiety [107-109] calls into question the value of disease-specific approaches.

The studies in this review also proposed additional tailoring factors for future consideration. In a qualitative investigation, Igelström et al [80] recommended further tailoring of DHI information, design, and features to the individual with regard to their treatment as well as other factors that appeared to affect the user’s experience such as computer experience, internet activity, and interactions with social media. In another study on the same platform [77], patients desired further tailoring to their specific diagnosis, age, sex, treatment, and symptoms, despite the DHI being tailored to the condition (cancer). For the PeerTECH intervention, certified peer specialists personalized SMS text messages on topics such as stories of recovery, medication adherence, coping skills training, and sleep hygiene [50]. Others have raised the possibility of tailoring to participants’ preconceptions of the interventions, such as meditation [97] and symptom tracking [80]. Thus, although tailoring can be an important component of DHIs for this population, whether tailoring should be to the *person* instead of the *condition* requires further investigation.

#### Guidance

Interventions reported among the included studies were predominately guided; however, both guided and self-guided DHIs were represented among studies reporting effective outcomes, suggesting that outcomes may not be compromised without guidance. The qualifications of individuals providing guidance varied considerably (eg, laypersons to psychiatrists), as did their training and role in the intervention. Similar findings regarding the heterogeneity in the guidance of internet-based CBT for chronic conditions have been reported previously by a meta-analysis by van Began et al [30]. Such variability may arise because of confusion regarding whether guidance constitutes the intervention itself or the supporting context (promoting engagement with technology or study procedures). Indeed, although several interventions relied on guidance to promote engagement with the intervention [50,55,57,60,64,91,99], studies have noted that this limited the scalability of the intervention. Guidance provided by lay individuals and peer supporters was feasible and well accepted [46,60], whereas interventions supported by nurses at times faced challenges in integrating additional workload into their roles [90]. Although several alternatives exist to improve the feasibility of guidance (eg, reduced frequency of guidance, partial guidance, no guidance, blending guided sessions with self-help modules [110], and automated guidance via chatbots [80]), the findings of this review and previous studies underscore the potential for allied health professionals and nonclinicians in guiding DHIs. Nevertheless, clarity is needed regarding the time spent by these individuals and the nature of the support provided (ie, technical support or health-related support) to understand how to support DHIs safely and effectively for this population.

#### Intensity

Given the complexity of the population with chronic conditions and comorbid depression or anxiety, identifying for whom DHIs are most appropriate was a significant line of investigation [88,94,98]. Among the studies included in this review, those with more severe depressive symptoms often benefited most from DHI treatment. Puzia et al [94] found that patients with myeloproliferative neoplasms with the poorest baseline global mental health experienced the largest reductions in depression and anxiety symptoms when using a calm mindfulness meditation app. Similarly, despite finding no significant differences in anxiety between a tailored CBT app and control, Greer et al [79] reported that patients with more severe anxiety at baseline benefited the most from the CBT app. Although it is commonly accepted that those with more severe mental health symptoms warrant more intensive treatment [111,112], the findings of this review and previous studies suggest that these individuals also benefit from DHIs [113-115]. Taken together, these findings indicate that individuals with severe symptoms of depression or anxiety can benefit from interventions of varying intensity, especially when intensity is defined by clinician time and contacts. It should be noted that the qualitative findings of the included studies reported that participants found DHIs to be demanding in terms of the time and skills required to read the intervention content and completing exercises in addition to the work involved in learning a new platform and troubleshooting technical and navigation issues [47]. Therefore, an expanded conceptualization of intervention intensity may be fruitful in not only considering intervention intensity in terms of health system resources (eg, clinician time and involvement) but also the patient work involved.

#### Stepped Care

Presumably, resolving the mixed findings regarding the previously mentioned factors may not require standardizing the same level of guidance and tailoring for all interventions. It is possible that a variety of DHIs may be delivered and supported through a spectrum of guidance ranging from lay individuals to psychiatrists, with guidance tailored and intensified based on individual needs and preferences. This approach is consistent with a stepped care model that was envisioned to benefit from DHIs in several of the included studies [57,65,77,80,98] and previous reviews on this topic [28,35]. Although over a decade has passed since Cuijpers et al [28] first envisioned the role of DHIs for this population existing within stepped care models, this review identified that the application of these interventions in stepped care models has only recently begun. Nevertheless, the emerging work by Igelström et al [80] demonstrates the promise of this approach in their iCAN-DO stepped care model for patients with cancer, with depression or anxiety symptoms, which included nurse-led (step 1, psychoeducation) and psychologist-led (step 2, internet-based CBT) DHIs. As most patients (60%) in the study did not use the second step of support, a stepped care approach may allow for efficient use of scarce mental health human resources [80]. Early findings on the iCAN-DO stepped care intervention indicated promising results regarding the efficacy of this intervention with reductions in depressive symptoms in some patients with cancer [116]. However, more definitive research is needed to determine whether individuals with single and multiple chronic conditions [57] can benefit from stepped care models, particularly those delivered digitally.

### Limitations

This review had several limitations. First, this review was limited to DHIs for depression or anxiety among people with one or more common chronic conditions identified by the Public Health Agency of Canada [43]. Although not included in this review, other chronic conditions not within the scope of this study (chronic pain, irritable bowel syndrome, tinnitus, epilepsy, etc) have been investigated in previous syntheses [28-32,35,37]. Second, despite engaging a discipline-specific research librarian when developing the search strategy and using multiple search methods (eg, hand searching and reviewing reference lists), the search strategy may have missed relevant studies. Third, because of our interest in DHIs that leverage newer technologies, studies that relied solely on phone calls were excluded. Thus, interventions that may be beneficial, such as telephone-based counseling [117-119] and automated telephone screening [120,121] were not within the scope of this study. As studies with older adults with chronic conditions have identified telephone-based support as a desirable component of mental health support [122], further research is needed on the use of phone calls. Fourth, the restriction to studies that were published in peer-reviewed journals in the English language may have missed publicly available DHIs (eg, mobile apps in app stores) that have not yet been researched or limited the inclusion of studies from non–English-speaking countries. Fifth, to be included, studies in this review must have recruited participants with co-occurring depression or anxiety with chronic conditions or explicitly stated that the goal of the DHI was to prevent, detect, or treat depression or anxiety among people with one or more chronic conditions. Broader aspects of mental health (ie, psychological distress [123], social support [124]), general self-management programs [125], and other mental health conditions (ie, eating disorders and substance use disorders) were beyond the scope of this study and warrant investigation [126].

### Conclusions

Amidst meta-analytic research documenting the potential benefits of DHIs to address depression or anxiety among people living with chronic conditions, this scoping review addresses the paucity of research focusing on the design and implementation considerations of such interventions. This review found that the use of DHIs for depression or anxiety among individuals with chronic conditions is a rapidly growing area of research, with most interventions seeking to provide depression treatment using DHIs that are web based, guided, and tailored to chronic physical conditions. With few studies conducted to date, stepped care models are a promising model to implement efficacious DHIs into standard care, although more definitive research is needed to determine whether individuals with single and multiple chronic conditions can benefit from these models. In constructing such models, questions regarding DHI guidance, tailoring, and intensity are key considerations and require future research. Developments in these areas will aid in realizing the potential of DHIs to transform care for patients with chronic conditions consistent with their holistic health needs.

## Appendix

Multimedia Appendix 1 PRISMA-ScR (Preferred Reporting Items for Systematic Reviews and Meta-Analyses extension for Scoping Reviews) checklist.

Multimedia Appendix 2 Sample search strategy (MEDLINE).

Multimedia Appendix 3 Summary of digital health interventions.

Multimedia Appendix 4 Summary of digital health intervention components.

Multimedia Appendix 5 Summary of digital health intervention guidance.

## Abbreviations

CBT: cognitive behavioral therapy
DHI: digital health intervention
PRISMA-ScR: Preferred Reporting Items for Systematic Reviews and Meta-Analyses extension for Scoping Reviews
