# Supplementary material for: Digital Health Interventions for Depression and Anxiety Among People With Chronic Conditions: Scoping Review
Source: J Med Internet Res. 2022 Sep 26;24(9):e38030. doi: 10.2196/38030 (PMC9555324; doi:10.2196/38030)
Supplement: Multimedia Appendix 2 [file jmir_v24i9e38030_app2.docx]

Multimedia Appendix 2. Search Strategy for MEDLINE (Ovid platform)

1. exp Comorbidity/
2. (comorbid$ or co morbid*).ti,ab.
3. (coocur* or co occur* or coexist$ or co exist$ or concomitant).ti,ab.
4. or/1-3
5. exp Multimorbidity/
6. (multimorbid$ or (multi morbid) or (multi morbidity) or (multi morbidities)).ti,ab.
7. (multidisease? or (multi disease) or (multi diseases) or (polypathology)).ti,ab.
8. exp Multiple Chronic Conditions/
9. (multiple adj3 (ill or ills or illness$ or disease? or condition? or syndrom* or disorder* or health* or medication* or symptom* or care)).ti,ab
10. ((complex care) or (complex need*) or (complex care need*) or (complex chronic disease and disability) or (long term)).ti,ab.
11. or/5-10
12. exp Chronic Disease/
13. (chronic$ adj3 (disease? or ill or ills or illness$ or care or condition? or disorder$ or syndrom$ or symptom$ or health$ or medication$)).ti,ab.
14. or/12-13
15. 4 or 11 or 14
16. exp diabetes mellitus/
17. diabet$.ti,ab.
18. exp hypertension/
19. (hypertens$ or "high blood pressure?").ti,ab.
20. exp heart diseases/
21. (((heart or cardiac or cardiovascular or coronary) adj (disease? or disorder? or failure)) or arrythmia?).ti,ab.
22. exp cerebrovascular disorders/
23. ((cerebrovascular or vascular or carotoid$ or arter$) adj (disorder? or disease?)).ti,ab.
24. exp asthma/
25. asthma$.ti,ab.
26. exp pulmonary disease chronic obstructive/
27. (copd or (pulmonary adj2 (disease? or disorder?))).ti,ab.
28. exp arthritis rheumatoid/
29. (rheumatoid arthritis).ti,ab.
30. exp neoplasms/
31. (neoplasm? or cancer?).ti,ab.
32. exp Alzheimer Disease/
33. alzheimer*.ti,ab.
34. or/16‐33
35. ((coocur$ or co ocur$ or coexist$ or co exist$ or multipl$) adj3 (disease? or ill$ or care or condition? or disorder$ or health$ or medication$ or symptom$ or syndrom$)).ti,ab.
36. chronic$.ti,ab.
37. 35 or 36
38. exp Mental health/
39. (mental health).ti,ab.
40. exp Psychology/
41. (psycholog* or psychosocial or biopsychosocial).ti,ab.
42. psychiatric.ti,ab.
43. (emotional health or emotion*).ti,ab.
44. (stress or distress or (psychological distress)).ti,ab.
45. exp Depression/
46. depression.ti,ab.
47. exp Depressive Disorder/
48. ((depressive disorder) or (depressive disorders) or (depressive symptom) or (depressive symptoms)).ti,ab.
49. exp Anxiety/
50. (anxiety or (anxiety disorder) or (anxiety disorders)).ti,ab.
51. exp Psychosomatic Medicine/
52. psychosomatic.ti,ab.
53. or/38-52
54. exp Telemedicine/
55. (Telehealth or (tele health) or telecare or telematics or teleconsultation or telemonitoring or telepresence or teleintervention or (tele intervention) or (health telematics)).ti,ab.
56. ((remote monitoring) or (self monitoring) or (home monitoring) or (home health monitoring)).ti,ab.
57. (SMS or telephone or texting or smartphone or android or app or apps or cellphone or (cell phone) or iphone or iPhone).ti,ab.
58. (mHealth or mhealth).ti,ab.
59. (eHealth or ehealth or etherapy or e-rehabilitation).ti,ab.
60. ((emental health*) or (e mental health*) or (e therap*) or (e psycholog*) or cybertherap* or cyberpsycholog*).ti,ab.
61. exp Medical Informatics/
62. (medical informatics or health informatics).ti,ab.
63. exp Public Health Informatics/
64. (public health informatics).ti,ab.
65. exp Consumer Health Informatics/
66. ((consumer health informatics) or (consumer health application)).ti,ab.
67. exp Patient Portals/
68. ((patient portal) or (patient portals)).ti,ab.
69. (e-Portal* or ePortal* or (electronic health information)).ti,ab.
70. (ICT or (information communication technology)).ti,ab.
71. ((health information technology) or (healthcare technology)).ti,ab.
72. exp Electronic Health Records/
73. ((electronic health records or EHR)).ti,ab.
74. exp Internet/
75. (web or website* or internet* or forum* or blog or computer or cyber* or online* or online or wireless).ti,ab.
76. (digital health or digital* or technolog*).ti,ab.
77. (ipad* or i-pad*).ti,ab.
78. or/54-77
79. 15 or 34 or 46
