# Supplementary material for: Digital Health Interventions for Depression and Anxiety Among People With Chronic Conditions: Scoping Review
Source: J Med Internet Res. 2022 Sep 26;24(9):e38030. doi: 10.2196/38030 (PMC9555324; doi:10.2196/38030)
Supplement: Multimedia Appendix 3 [file jmir_v24i9e38030_app3.docx]

| Multimedia Appendix 5: Summary of DHI Guidance | | | | |
| --- | --- | --- | --- | --- |
| 1^st^ Author, year | **Source** | **Method** | **Purpose** | **Frequency** |
| Ali, 2020 [73] | Care coordinator | Phone or in-person | Symptom monitoring, counselling patient towards goals | Every 2-4 weeks |
| Arch, 2020 [55] | Oncology social worker and psychologist | Online & in-person | Online check-ins and co-lead manualized in-person group sessions | Online check-ins: daily, 5-7 mins  In-person group sessions: 4 x 2-hour group sessions |
| Brandt, 2019 [91] | Nurse | In-person & phone calls | Training to use the device, monitoring progress through intervention, and positive reinforcement | 45-60 min for initial training, 15-min follow up calls, and 15-30 min for last appointment |
| Chun, 2020 [60] | Stroke physician trained in CBT | Telephone | Counselling | Weekly for 6 weeks, 35-45 minutes each |
| Cohen, 2020 [96] | Pharmacist | In-person and through telehealth equipment/phone | Training to use the device/intervention, monitoring symptoms, providing positive reinforcement/encouragement, answering questions, providing information and feedback | 2 in-person visits (one at baseline and the other at 6 months follow-up) followed by daily monitoring and connecting with patients as needed via phone and telehealth system should symptoms change |
| Druss, 2014 [59] | Study staff | In-person | Computer literacy training and intervention orientation | 4-hour training curriculum; 1 hour visit followed by 30 min visits as needed |
| Fortuna, 2018a [61] | Certified Peer Specialists | Text message and in-person, at home sessions | Support and promote engagement | Text messages 3x/week; weekly in-person sessions |
| Fortuna, 2018b [51] | Certified Peer Specialists | Text message and in-person, at home sessions | Support and promote engagement | Text messages 3x/week; weekly in-person sessions |
| Fortuna, 2018c [65] | Certified Peer Specialists | Text message and in-person, at home sessions | Support and promote engagement | Text messages 3x/week; weekly in-person sessions |
| Fortuna, 2019 [56] | Certified Peer Specialists | Text message and in-person, at home sessions | Support and promote engagement | Text messages 3x/week; weekly in-person sessions |
| Hauffman, 2017 [87] | Nurse | Online | Answer questions and moderate forum | Unclear |
| Hauffman, 2020a [78] | Nurse (step 1) and psychologist (step 2) | Online via portal | Answer questions and moderate forum (step 1, nurse)  Monitor progress, answer questions, and prvide feedback (step 2, psychologist) | Step 1: Unclear  Step 2: Weekly |
| Hauffman, 2020b [82] | Nurse (step 1) and psychologist (step 2) | Online via portal | Answer questions and moderate forum (step 1, nurse)  Monitor progress, answer questions, and provide feedback (step 2, psychologist) | Step 1: Unclear  Step 2: Weekly |
| Igelstrom, 2020 [81] | Nurse (step 1) and psychologist (step 2) | Online via portal | Answer questions and moderate forum (step 1, nurse)  Monitor progress, answer questions, and provide feedback (step 2, psychologist) | Step 1: Unclear  Step 2: Weekly |
| Lundgren, 2015 [48] | Nurse specialist | Email | Answer questions and provide reminders | Within 24 hrs on weekdays |
| Lundgren, 2016 [66] | Nurse specialist | Email | Answer questions and provide reminders | Within 24 hrs on weekdays |
| Johansson et al. 2021 [93] | Nurse specialist | Email | Respond to questions and provide reminders | Within 24 hrs on weekdays |
| Koehler, 2020 [72] | Nurse | Phone | Structured interview including questions about mood | Monthly or as requested/needed by the patient |
| McCusker, 2015 [47] | Trained lay coach | Phone | Monitor symptom severity and tailor intervention to individual needs | Weekly for 3 months, monthly for 6 months |
| McCusker, 2016 [79] | Trained lay coach | Phone | Monitor symptom severity and tailor intervention to individual needs | Weekly for 3 months, monthly for 6 months |
| Menezes, 2019 [47] | Nurse or nurse assistant | Phone calls | Training to use the device, monitoring progress through intervention, and positive reinforcement | After weeks 1 and 4 |
| Newby, 2017 [92] | Clinical psychologists or psychiatrist registrars | Email and phone | Support and promote engagement | After lesson 1 & 2; as required after |
| Nobis, 2015 [100] | Graduate student or psychologist | Email and text messages or phone calls | Feedback, promote adherence | Email feedback 48 hrs after session; text messages/calls frequency unclear |
| Read, 2020 [58] | Psychologist | Phone or email | Promote engagement, answer questions, and provide encouragement | Weekly |
| Schlicker, 2019 [99] | Psychologist | Email and text messages or phone calls | Provide feedback and reminders | Email feedback 48 hours after session; text messages/calls frequency unclear |
| Sorocco, 2013 [67] | CCHT providers | Unclear | Unclear | Unclear |
| Steel, 2011 [85] | Nurse and doctoral-level psychologists | Phone calls | Unclear | Min 2 (before and after treatment) |
| Stevenson, 2019 [97] | Nurse | Phone call (patient initiated or requested through  Web-based tool) | Answer questions and provide/supplement information in the web-based tool | As needed |
| Sui, 2019 [71] | Nurse | WeChat messages (group and 1:1) | Support and scheduling appointments | Weekly and as needed |
| van Bastelaar, 2011 (a) [75] | Case psychologists | Email | Homework feedback and assignment reminders | Within 3 working days |
| van Bastelaar, 2011 (b) [53] | Case psychologists | Email | Homework feedback and assignment reminders | Within 3 working days |
| van Bastelaar, 2012 [89] | Case psychologists | Email | Homework feedback and assignment reminders | Within 3 working days |
| Wilson, 2018 [63] | Psychiatric nurse practitioners and psychiatrist | Email or phone | Promote engagement and answer questions. Additional troubleshooting assistance available upon request | Weekly and help as needed |
