# Supplementary material for: Digital Health Interventions for Depression and Anxiety Among People With Chronic Conditions: Scoping Review
Source: J Med Internet Res. 2022 Sep 26;24(9):e38030. doi: 10.2196/38030 (PMC9555324; doi:10.2196/38030)
Supplement: Multimedia Appendix 4 [file jmir_v24i9e38030_app4.docx]

Multimedia Appendix 4: DHI Components

| **1^st^ Author, Year** | **Tailored**  **(Condition)** | **Guided** | **Education** | **Psychological Therapy** | **Peer Support** | **Communication**  **with HCP** | **Mindfulness** | **Chat Room/**  **Forum** | **Monitoring of MH Status** |
| --- | --- | --- | --- | --- | --- | --- | --- | --- | --- |
| Ali, 2020 [73] |  | ✓ |  |  |  | ✓ |  |  | ✓ |
| Arch, 2020 [55] | ✓ |  |  | ✓ (ACT) |  |  |  |  |  |
| Bajracharya, 2016 [77] |  |  |  |  |  |  |  |  |  |
| Baldwin, 2020 [50] |  |  | ✓ | ✓ (CBT) |  |  |  |  | ✓ |
| Brandt, 2019 [91] |  | ✓ | ✓ | ✓ (BA) |  |  |  |  |  |
| Chun, 2020 [60] | ✓ | ✓ | ✓ | ✓ (CBT) |  |  |  |  |  |
| Clarke, 2016 [64] |  |  | ✓ | ✓ (CBT) |  |  |  |  | ✓ |
| Clarke, 2019 [94] |  |  | ✓ | ✓ (CBT) |  |  |  |  | ✓ |
| Cohen, 2020 [96] |  | ✓ |  |  |  | ✓ |  |  |  |
| Druss, 2014 [59] |  | ✓ |  |  |  |  |  |  |  |
| Fletcher, 2019 [86] |  |  | ✓ | ✓ (CBT) |  |  |  |  | ✓ |
| Fortuna, 2018a [61] |  | ✓ | ✓ |  | ✓ |  |  |  |  |
| Fortuna, 2018b [51] |  | ✓ | ✓ |  | ✓ |  |  |  |  |
| Fortuna, 2018c [65] |  | ✓ | ✓ |  | ✓ |  |  |  |  |
| Fortuna, 2019 [56] |  | ✓ | ✓ |  | ✓ |  |  |  |  |
| Greer, 2019 [80] | ✓ |  |  | ✓ (CBT) |  |  |  |  |  |
| Hauffman, 2017 [87] | ✓ | ✓ | ✓ |  | ✓ | ✓ |  | ✓ |  |
| Hauffman, 2020 [78] | ✓ | ✓ | ✓ | ✓ (CBT) | ✓ | ✓ |  | ✓ |  |
| Hauffman, 2020 [82] | ✓ | ✓ | ✓ | ✓ (CBT) | ✓ | ✓ |  | ✓ |  |
| Huberty, 2019 [88] |  |  |  |  |  |  | ✓ |  |  |
| Huberty, 2019 [98] |  |  |  |  |  |  | ✓ |  |  |
| Igelström, 2020 [81] | ✓ | ✓ | ✓ | ✓ (CBT) | ✓ | ✓ |  | ✓ |  |
| Jin, 2020 [83] |  |  |  |  |  |  |  |  | ✓ |
| Jindal, 2018 [62] |  |  |  |  |  |  |  |  |  |
| Johansson, 2021 [93] | ✓ | ✓ |  | ✓ (CBT) |  |  |  |  |  |
| Kayyali, 2016 [57] |  |  |  |  |  |  |  |  | ✓ |
| Kearney, 2021 [49] | ✓ |  |  |  |  | ✓ |  |  | ✓ |
| Koehler, 2020 [72] |  | ✓ |  |  |  | ✓ |  |  |  |
| Lundgren, 2015 [48] | ✓ | ✓ |  | ✓ (CBT) |  |  |  |  |  |
| Lundgren, 2016 [66] | ✓ | ✓ |  | ✓ (CBT) |  |  |  |  |  |
| McCusker, 2015 [79] |  | ✓ | ✓ | ✓ (CBT) |  |  |  |  | ✓ |
| McCusker, 2016 [84] |  | ✓ | ✓ | ✓ (CBT) |  |  |  |  | ✓ |
| Menezes, 2019 [69] |  | ✓ | ✓ | ✓ (BA) |  |  |  |  |  |
| Mohammad, 2019 [52] |  |  |  |  |  |  |  |  |  |
| Newby, 2017 [92] |  | ✓ |  | ✓ (CBT) |  |  |  |  |  |
| Nobis, 2015 [100] | ✓ | ✓ | ✓ | ✓ (BA, PS) |  |  |  |  |  |
| Prabhakaran, 2019 [76] |  |  |  |  |  |  |  |  |  |
| Puzia, 2020 [95] |  |  |  | ✓ (CBT) |  |  | ✓ |  |  |
| Read, 2020 [58] |  | ✓ | ✓ | ✓ (CBT) |  |  |  |  |  |
| Sarda, 2019 [74] |  |  |  |  |  |  |  |  | ✓ |
| Schlicker, 2019 [99] | ✓ | ✓ | ✓ | ✓ (BA, PS) |  |  |  |  |  |
| Sorocco, 2013 [67] |  | ✓ |  | ✓ |  |  |  |  | ✓ |
| Steel, 2011 [85] |  | ✓ | ✓ |  | ✓ |  |  | ✓ |  |
| Stevenson, 2020 [97] | ✓ | ✓ | ✓ |  |  |  |  | ✓ |  |
| Sui, 2020 [71] | ✓ | ✓ | ✓ | ✓ |  |  |  | ✓ |  |
| van Bastelaar, 2011 (a) [75] | ✓ | ✓ | ✓ | ✓ (CBT) |  |  |  |  |  |
| van Bastelaar, 2011 (b) [53] | ✓ | ✓ | ✓ | ✓ (CBT) |  |  |  |  |  |
| van Bastelaar, 2012 [89] | ✓ | ✓ | ✓ | ✓ (CBT) |  |  |  |  |  |
| Whelan, 2019 [68] | ✓ |  | ✓ |  |  |  |  |  | ✓ |
| Whiteman, 2017 [70] |  |  | ✓ |  |  | ✓ |  |  |  |
| Wilson, 2018 [63] |  | ✓ | ✓ | ✓ (CBT) |  |  |  |  | ✓ |
| Wittink, 2017 [90] |  |  |  |  |  |  |  |  |  |
| Ye, 2020 [54] |  |  | ✓ |  |  |  |  |  |  |

ACT: Acceptance and Commitment Therapy

BA: Behavioural Activation

CBT: Cognitive Behavioural Therapy

MH: Mental Health
