# Supplementary material for: Digital Health Interventions for Depression and Anxiety Among People With Chronic Conditions: Scoping Review
Source: J Med Internet Res. 2022 Sep 26;24(9):e38030. doi: 10.2196/38030 (PMC9555324; doi:10.2196/38030)
Supplement: Multimedia Appendix 5 [file jmir_v24i9e38030_app5.docx]

Multimedia Appendix 3: Summary of DHIs

| 1^st^ Author, Year | Study Phase  (Sample Size) | Depression/Anxiety | Chronic Conditions | Digital Technology | Intervention Description | Main findings |
| --- | --- | --- | --- | --- | --- | --- |
| PREVENTION | | | | | | |
| Wittink, 2017 [90] | Effectiveness  (n=60) | Depression & anxiety | Any chronic condition | Web-based | **Customized Care:** Web-based platform designed to assist patients in stressor disclosure to PCP by addressing barriers to patient-centered communication. Consists of computer-based Discussion Prioritization Tool to assist patients in prioritization of their stressors followed by an automatically generated question prompt list that is printed for patients to share during their PCP visit. | Patients receiving Customized Care were significantly more likely to disclose stressors and reported greater confidence in stressor disclosure. No significant differences in patient activation or length of office visit between intervention and usual care control. |
| Stevenson, 2020  [97] | Effectiveness  (n=60) | Depression & anxiety | Cancer | Web-based | **Web-based Information Tool:** Web-based platform with information tailored to cancer (for both patients and caregivers); relevant website links; and a discussion forum for participants to interact. Information was based on local resources (e.g., Leukemia Foundation of Australia), refined using a multidisciplinary advisory group, and could be tailored by the clinician at time of enrollment to the specific cancer diagnosis and treatment options. Intervention designed to be accessed at time of diagnosis and throughout the course of treatment. | No significant differences in unmet information needs, depression, or anxiety between the intervention group (web-based information tool with nurse phone support) and control. Findings were inclusive as the study was underpowered due to recruitment challenges. |
| Read, 2020 [58] | Efficacy  (n=302) | Depression | Multimorbidity (2 or more conditions) | Computer and tablet | **iCBT:** Based on the Wellbeing Plus Course designed for individuals aged 60 and older that is freely available in Australia. Consists of 5 CBT lessons delivered over 8 weeks. | iCBT prevented depressive disorder among older adults with multimorbidity without clinically significant depressive symptoms in the first six months post-intervention. Cases of depressive disorder were three times higher in the control group than the iCBT group. No significant differences were observed in secondary outcomes such as anxiety, pain, and disability. |
| DETECTION | | | | | | |
| Bajracharya, 2016  [77] | Feasibility, acceptability, and effectiveness  (n=378) | Depression | Diabetes (T2) | EHR | **Unnamed:** Incorporation of depression screening tool (PHQ-2 & PHQ-9) in patient’s EHR and implementation of depression screening protocol to providers. EHR automatically prompts provider to ask questions on PHQ-9 for patients screening positive on the PHQ-2, with PHQ-9 scores ≥5 warranting intervention by the provider. | Implementation of brief and valid screening tools (PHQ-2 and PHQ-9) into EHR alongside a screening protocol for providers significantly increased screening of depression during primary care visits. |
| Kayyali, 2016  [57] | Feasibility & acceptability (n=32) | Depression & anxiety | COPD | Wearable and mobile phone | **Wearable Sensing and Smart Cloud Computing for Integrated Care to COPD Patients with Co-morbidities System (WELCOME):** Telehealth system consisting of (1) wearable vest with sensors to monitor physiological measurements with patient-held devices (e.g., blood glucose metre, inhaler adherence monitoring device); and (2) a mobile phone app for mental health assessment and lifestyle advice. | Patients, health care providers, and informal carers perceived the WELCOME system positively due to the sense of safety it provided. Suggestions for improvement included personalizing frequency and duration of monitoring to the patient; triaging alerts through a “traffic light” system; and providing information through text messages instead of through an app. |
| Sarda, 2019  [74] | Evaluate prediction model  (n=47) | Depression | Diabetes (T1 & T2) | Mobile phone | **Unnamed:** Smartphone sensing app for family members to remotely and non-intrusively care for and check on their family member’s wellbeing. Every 2 minutes, app passively collects information related to activity rates (via accelerometer and GPS); communication levels (via call logs); sleep patterns (via accelerometer and ambient light sensor); and mobility (via GPS). App includes machine learning platform to assist in detecting probable changes in wellbeing. | Individuals with diabetes and self-reported symptoms of major depression displayed lower levels of social contact and activity during the day. Predictive modeling using smartphone sensing information may be a promising method for the early detection of depressive symptoms in people with diabetes. |
| Whelan, 2019  [68] | Adherence & use  (n=107) | Depression & anxiety | COPD | Mobile device | **sElf-management anD support proGrammE (EDGE):** Self-management system including (1) COPD symptom questionnaire; (2) a Bluetooth-enabled pulse oximeter; (3) multimedia content; and (4) mood monitoring (PHQ-2 and GAD-2 with no tracking of scores over time) 2 weeks after starting the system and then at 4-week intervals. Those scoring >1 were prompted to complete the full PHQ-8 or GAD-7. Individuals with scores ≥5 on either tool were signposted (automatically directed) to either or both stress (~3.5 mins) or mood management (~1.5 mins) videos. Clinician reviewed data at 4-day intervals and PCPs were notified by letter of PHQ-8/GAD-7 scores ≥10. | Monitoring of anxiety and depressive symptoms as part of a multicomponent digital health system for COPD was feasible and acceptable. A high rate of adherence to the monthly mood monitoring was observed, with some participants entering multiple responses each month. Over half of the trial participants reported at least one instance of clinically significant symptoms of anxiety and depression during the 12-month study period. Signposting videos to participants who reported elevated symptoms increased the viewing time of videos. |
| Jin, 2020 [83] | Psychometric  (n=206) | Depression | Diabetes | Mobile device | **SMS Text Messaging:** Screening for depression (PHQ-2/PHQ-8), anxiety (GAD-2), and functional disability (SDS) delivered through SMS text messaging in English or Spanish as selected by the participant. | Depression screening using the PHQ-8 via SMS demonstrated good internal consistency, test-retest reliability, and concordance while shorter scales (PHQ-2, GAD-2, and SDS) had poor to moderate levels of concordance between the SMS and interview assessment modes. Participants reported more depressive and anxiety symptoms through SMS compared to interview assessments, suggesting that SMS offers a discrete option with less stigma and promotes self-disclosure of symptoms. Personality traits and demographic factors were associated with differences between the SMS and interview assessment modes. |
| TREATMENT | | | | | | |
| Steel, 2011 [85] | Efficacy  (Sample size not specified) | Depression | Cancer | Web-based | **Unnamed:** Website as part of a multi-component stepped collaborative care intervention including face-to-face visits and follow-up phone calls by care coordinator (min. 2, frequency stepped as needed). Website included (1) educational information; (2) self-management area; (3) journaling; (4) chat room; (5) an audiovisual library; and (6) peer support. Website designed with larger font and a text-to-voice option to improve accessibility to older and low literate individuals. | Use of collaborative care intervention to manage multiple symptoms within a tertiary oncology centre is feasible. Issues remain related to the intervention development for the oncology context; use of website for populations with chronic conditions; evaluating intervention efficacy; and interpretation of collaborative care interventions. |
| van Bastelaar, 2011 (a) [75] | Design & development  (n=255) | Depression | Diabetes (T1 & T2) | Web-based | **Coping with Depression ('Kleur je Leven'):** Self-help course adapted from a manual-based course and further tailored to needs of people with diabetes (diabetes-specific information added to the intervention). Consists of 8 CBT lessons with information, practice examples, exercises/self-tests, and homework assignments with weekly protocolized e-mail feedback from coaches. Moderated internet-based support forum available for participants to share experiences, provide support and discuss issues. | A diabetes-specific version of an existing effective web-based program for depression attracted interest of a large patient group (540), with 80% of patients reporting a diabetes-specific approach as important for enrollment. Adding diabetes-specific content within depression treatment may serve as a model for self-help interventions for other conditions. |
| van Bastelaar, 2011 (b) [53] | Effectiveness  (n=255) |  |  |  |  | Coping with Depression was effective in reducing symptoms of depression and diabetes-specific emotional distress for patients with type 1 or type 2 diabetes but had no significant effects on glycemic control. |
| van Bastelaar, 2012 [89] | Secondary analysis  (n=255) |  |  |  |  | Diagnosis of MDD, anxiety disorder, or elevated diabetes distress did not significantly modify the effect of web-based diabetes-specific CBT for individuals with self-assessed depressive symptoms. Diabetes-specific web-based CBT is suitable for both individuals with mild depressive symptoms as well as those with a more severe clinical profile. |
| Sorocco, 2013 [67] | Feasibility & acceptability  (n=6) | Depression & anxiety | Any chronic condition | Video telehealth and remote monitoring | **LifeView System:** System combining video telehealth and remote monitoring of vitals and symptoms. Patients can access psychotherapy by a psychologist or routine medical care via interactive video or audio telehealth visits. Monitoring of daily vitals by Care Coordination Home Telehealth providers is enabled by peripheral tools (digital scale, blood pressure monitor, pulse oximeter, digital stethoscope, and glucose meter). | Integration of Care Coordination Home Telehealth and televideo (LifeView) within home-based primary care appears to be a feasible and clinically beneficial method to expand medical care for rural veterans classified as medically complex. |
| Druss, 2014 [59] | Effectiveness  (n=170) | Depression | Any chronic condition | Web-based | **My Health Record:** Adapted an existing community-based personal health record to help patients with chronic medical conditions self-manage and interact with the health system. Consists of (1) personal details; (2) diagnoses; (3) goals and action steps; (4) health indicators (i.e., blood pressure, glucose level); (5) medications and allergies; (6) hospital visits; (7) immunizations; and (8) health and family health history. Program was adapted to be at a sixth grade reading level and to include section for mental health and health goals, preferences about mental health care in case of incapacitation, and information about community resources. Patients receive prompts as reminders for routine preventive services. | Having a personal health record was associated with higher rates of medical service utilization, and improved quality of preventative and cardiometabolic care, but no benefits on patient activation, quality of life, or other indicators of service use. |
| Lundgren, 2015 [48] | Design, development, feasibility, and acceptability  (n=7) | Depression | Heart failure | Web-based | **ICBT**: Guided web-based program, consisting of 7 modules across 9 weeks. Each module consists of text (living with HF, problem-solving, behaviour activation) and exercises with written feedback provided by a mental health nurse specialist via secure email system. Personalized, manually written emails sent to those who did not complete modules. | ICBT was feasible and time efficient. Median depressive symptoms decreased in all patients at the end of the treatment, with no patients scores worsening. Feedback required ~3 hours of health care provider time per patient. Patients perceived ICBT as facilitative by allowing them to learn about their health problems/strategies, allowing them the time to learn, and offering the freedom to learn alongside their other life obligations. Patients also perceived the platform as demanding due to technical problems, or by requiring reading and work. |
| Lundgren, 2016 [66] | Effectiveness  (n=50) |  |  |  |  | No significant differences in depressive symptoms, cardiac anxiety, and quality of life between guided ICBT and a moderated discussion forum were observed post-treatment. However, statistically significant improvements in depressive symptoms were observed within the ICBT group. |
| Johansson, 2021 [93] | Secondary analysis  (n=50) |  |  |  |  | No significant differences in self-care among patients with heart failure and depressive symptoms between the ICBT and the online discussion control at 3- and 9-weeks follow-ups. |
| Nobis, 2015 [100] | Efficacy  (n=260) | Depression | Diabetes (T1 & T2) | Web-based with mobile phone support | **GET.ON Mood Enhancer Diabetes (GET.ON M.E.D):** A minimally guided web-based intervention consisting of 6 consecutive sessions with 2 optional sessions on weight management and improving sleep. Intervention is based on systematic behavioural activation and problem-solving with diabetic-specific content integrated into each session. Personalized feedback is provided by coaches after each session. Text message coach (standardized daily text messages) and phone calls/e-mails from coach as needed to promote adherence are optional. Optional booster session offered one month after the intervention. | Guided web-based intervention with diabetes-specific content, GET.ON M.E.D, was effective to reduce depressive symptoms and diabetes-specific emotional distress, compared to a brief, active, unguided, web-based control; program for depression. No significant differences in diabetes self-management were observed. |
| Schlicker, 2019 [99] | Secondary analysis  (n=253) |  |  |  |  | Disease-specific severity indicators (major depressive disorder at baseline, depression severity, and average blood glucose control) significantly predicted the treatment outcomes of GET.ON M.E.D. GET.ON M.E.D was effective in reducing depressive symptom severity for participants with lower and higher depressive symptom severity, suggesting that those with more severe symptoms who are not suicidal should not be excluded from guided online self-help interventions. |
| McCusker, 2015 [47] | Effectiveness  (n=223) | Depression | Any chronic condition | DVD, CD, and websites | **Toolkit:** Multicomponent intervention consisting of core tools (paper and audio versions of an CBT-based Antidepressant Skills Workbook; mood monitoring tool, informational DVD); supplemental tools (relaxation CD, information on medication misuse and emotional eating, self-help resources); and additional information (reading materials, internet sites, community groups, and a booklet on depression for family and friends). Relaxation CD based on progressive muscle relaxation techniques, DVD called “Finding a way out of depression” includes testimonials to normalize depression and provide hope, and the list of suggested websites provides information on depression. | Benefits of depression self-care tools with lay coaching appears to be short-lived, due to the statistically significant improvements in depressive symptoms observed in the short term (3-months) but not in the long-term (6-months). Individuals with moderate depression severity and high self-efficacy at baseline appeared to benefit most from the addition of lay coaching. |
| McCusker, 2016 [79] | Secondary analysis  (n=215) |  |  |  |  | The effectiveness of a self-care intervention with lay coaching effectiveness was modified by self-efficacy, but not activation, at baseline. |
| Clarke, 2016 [64] | Feasibility, acceptability, and effectiveness  (n=89) | Depression | Diabetes (T2) | Web-based | **myCompass:** Automated, personalized, generic, self-guided and -paced CBT program accessible on Internet-enabled devices (i.e., mobile phone, tablet, computer). Consists of: (1) symptom assessments; (2) 12 interactive skill-building educational modules with generic (not diabetes-specific), evidence-based content and homework; (3) self-monitoring based on 20 cognitive and/or behavioural variables; and (4) automated and tailored email feedback on use of the program. A tailing-off period (4-week) where only the symptom monitoring function is available. | High satisfaction of myCompass program by participants for its convenience, ease of use, and relevance. Significant and sustained improvements in depressive symptoms, anxiety, and diabetes-specific distress. |
| Fletcher, 2019 [86] | Feasibility & acceptability  (n=740) |  |  |  |  | Trial recruitment in general practice settings was difficult with time-, labour-, and cost-intensive efforts unsuccessful in promoting recruitment. Online recruitment resulted in more participants, with those recruited online largely similar to those recruited in general practice, suggesting minimal threats to internal validity from online recruitment. |
| Clarke, 2019 [94] | Effectiveness  (n=780) |  |  |  |  | No significant differences in social and occupational functioning, depressive symptoms, anxiety symptoms, or diabetes-specific distress between myCompass and online attention control at 3-months follow-up. |
| Baldwin, 2020 [50] | Efficacy  (n=780) | Depression & anxiety |  |  |  | Across the 12-month study period, significant improvements in work and social functioning were observed for the control group (Healthy Lifestyles intervention) compared to the myCompass intervention group across. By the end of the trial, all participants regardless of intervention reported improvements in mental health and diabetes at the end of the trial. |
| Newby, 2017 [92] | Efficacy  (n=106) | Depression | Diabetes (T1 & T2) | Web-based | **iCBT-MDD:** Consists of 6 generic, automated, cartoon-style web-based lessons CBT skills delivered over 10 weeks, with a 5-day minimum wait time between lessons. Downloadable homework document with assignments available after each lesson as well as extra resources, FAQs, and recovery stories from previous participants. Automated email reminders provided when lessons became available. Access to usual care allowed among participants receiving iCBT. | Generic iCBT-MDD was efficacious in reducing depressive symptoms, general anxiety, general distress, and diabetes-specific distress at post-treatment, with gains maintained at 3-months. No significant differences in physical health (HbA1c) were observed. |
| Whiteman, 2017 [70] | Design & development  (n=10) | Depression | Diabetes, Cardiovascular Disease, Hypertension, and/or COPD | Mobile device | **Unnamed:** Adapted the Integrated Illness Management and Recovery (I-IMR) program (10-module program delivered by a masters-level clinician and nurse for 8-10 months) for smartphone delivery. Adaptations included fewer words/page (300), lower literacy content (fourth grade level), reduced duration (3 months with option to extend), automatic module progression, and daily self-management checklist (personalizable by patient or clinician). Components included psychoeducation (3 min videos), behavioural tailoring (medication management learning module with the ability to enter medications and set reminders), relapse-prevention training (personalized relapse plan), and coping skills training (guided videos). Accompanying care management dashboard with EHR integration capabilities allows clinicians to send HIPAA-compliant messages to the patient, view daily self-management data for early intervention, and prevent disengagement. The original I-IMR program is based on the stress vulnerability model. | Middle-aged and older adults with serious mental illness and minimal technical abilities could participate in a user-centred design and adaptation process of a smartphone self-management intervention. High usability ratings and satisfaction was reported by middle-aged and older adults with serious mental illness. |
| Hauffman, 2017 [87] | Design, development, and acceptability  (n=39) | Depression & anxiety | Cancer | Web-based | **Nurse-led, Internet-based Learning and Self-care (NILS) program:** Web-based platform providing information, self-care support and psychosocial support, consisting of a library, forum (moderated by nurse), chat (moderated by nurse), FAQ section, and Ask an Expert section for participants to ask a nurse questions. Each content area in the library contains (1) a main lecture by a health care professional (choice of written and audiovisual formats) to normalize and provide guidance on each problem; (2) movies, slideshows, exercises, or written materials to elaborate on management strategies; and (3) links and suggested readings. Participant also have a section to enter their online notes and narratives which can be kept private or shared with other participants. Both the diary and forum sections have a feature to automatically detect and flag predefined words to detect severe depression and suicidality. Informed by Orem’s self-care deficit nursing theory, social cognitive theory, and social learning theory. | A collaborative approach between patients, clinicians, and researchers can be beneficial to develop an acceptable interactive health communication application to support the self-care of cancer. |
| Wilson, 2018 [63] | Feasibility, acceptability, and preliminary efficacy  (n=53) | Depression | Any chronic condition | Web-based | **Think Clearly About Depression:** Web-based depression symptom management program for individuals with mild/moderate symptoms of depression to learn about the role of thinking in the development and maintenance of depression. Consists of: (1) daily check-in to track mood; (2) calendar to schedule mood-enhancing activities and reminders; and (3) videos, interactive activities, lists, and homework to identify, challenge, and replace depressive thoughts with constructive thinking. Program guide and weekly email or phone call included to promote program engagement and for participants to ask questions. Program can serve as a standalone intervention or as a supplement to psychotherapy. | Improvements in depressive symptoms and self-efficacy between participants enrolled in Thinking Clearly About Depression and the control. Participant engagement and satisfaction with the program were high. |
| Fortuna, 2018a [61] | Feasibility, acceptability, and preliminary effectiveness  (n=10) | Depression | Diabetes, cardiovascular disease, hypertension, and/or COPD | Mobile device | **PeerTECH:** Tablet eModules used during 1-hour weekly in-person, peer-delivered self-management training for chronic physical and mental illness. Smartphone app used in between sessions to provide (1) daily self-management tasks; (2) peer-led videos to support self-management skill education; (3) medication reminders; (4) a pedometer; and (5) a HIPAA-compliant chat with peer specialists. Informed by social support, experiential knowledge, social learning, and social comparison theories. | Patients and peer specialists found PeerTECH feasible and acceptable. Statistically significant differences in psychiatric self-management were observed but improvements in hope, quality of life, medical self-management skills, empowerment, and self-efficacy to manage chronic conditions were non-significant. |
| Fortuna, 2018b [51] | Acceptability  (n=11) |  |  |  |  | Human support from peers may promote behaviour change in a peer-delivered, technology-based intervention for self-management of physical and mental illnesses. |
| Fortuna, 2018c [65] | Feasibility & acceptability  (n=11) |  |  |  |  | Certified peer specialists integrated a peer philosophy into PeerTECH by encouraging self-determination; guiding their practice using a bio-psycho-spiritual framework; sharing their lived experiences of self-management; personalizing text messages to reinforce self-management skills development; and identifying unmet needs and advocating for human rights. |
| Fortuna, 2019 [56] | Secondary analysis  (n=11) |  |  |  |  | During the 12-week intervention, consumers sent 141 text messages and certified peer specialists sent 215 text messages. Peer specialists provided a range of illness self-management support via text messaging including supporting health behavior change, employing self-management therapeutic techniques, facilitating engagement with the health technology, and providing peer support. |
| Jindal, 2018 [62] | Design & development  (n=631) | Depression | Hypertension & diabetes | Mobile device | **mWellcare:** Consists of a mobile health system (Android app) and web-based component. The app was used by primary care nurses and doctors for electronic storage and integration of longitudinal health records; automated guideline-recommended decision support; lifestyle interventions tailored to the patient clinical profile; automatic patient SMS reminders and alerts; data collection tool for remote quality assurance of programs; and export of healthcare data to healthcare systems in compliance with standards. The NCD nurse role using the mWellcare system was to enable task-sharing/shifting from the physician (patient registration, initial evaluation, assessment and long-term follow up). A web interface component allows for data viewing and extraction, report generation, and user management by the nurses. Initial evaluation with nurse produces a decision support record printout for the patient to consult the physician whose final decision is later entered into the app to generate lifestyle advice and behavioural change messaging. | The mWellcare intervention was designed using the Medical Research Council framework. The intervention was designed to address gaps in usual care through integrated management of chronic conditions, evidence-based decision support to physicians and other health workers, longitudinal health data collection, adherence support, and lifestyle advice. Pilot testing of the intervention identified implementation barriers related to the application, knowledge and skills of nurses, and the health system. |
| Prabhakaran, 2019 [76] | Effectiveness  (n=3698) |  |  |  |  | No significant differences in primary (systolic blood pressure, HbA1c) and secondary outcomes (fasting blood glucose, total cholesterol, predicted 10-year risk of cardiovascular disease, body mass index, depression, and tobacco and alcohol use) between mWellcare and enhanced usual care. |
| Sui, 2020 [71] | Acceptability, effectiveness, and implementation  (n=200) | Depression & anxiety | Cancer | Mobile phone | **WeChat app-based Education and Rehabilitation Program (WERP):** Comprehensive health education and rehabilitation program using the WeChat app with 4 components. (1) Disease-related education: distribution of disease-related health education manual, and later, invitation to a WeChat Group where the trained nurses would deliver educational courses (weekly short videos over 12 weeks) in the group. Participants are required to reply “received” after watching the videos. (2) Rehabilitation exercise guidance: short video courses involving nurse demonstrations of exercises in the WeChat group initiated from the 13^th^ week and delivered weekly for 40 weeks. Questions related to (1) and (2) or requests to schedule clinic appointments could be sent to nurses in the WeChat Group or a 1-1 chat. (3) Daily activity supervision: individualized daily walking plan tailored to patient's physical status, preferences, and treating physicians’ advice. Plan was measured by steps counted by WeChat Movement. Supervision, motivation (weekly), and plan adjustments made by nurses. (4) Psychological support: biweekly nurse-led counseling via WeChat video call based on a holistic (physical, functional, psychological, social, spiritual) assessment of life and mental status. | WERP significantly improved depression, anxiety, and quality of life compared to the control group for patients with non-small cell lung cancer after undergoing surgical resection. 12-month and 60-month loss to follow-up were lower in the WERP group compared to control, although, there was minimal benefit of WERP on survival compared to the control. |
| Brandt, 2019 [91] | Feasibility & acceptability  (n=35) | Depression | Diabetes (T1 & T2) and/or hypertension | Mobile phone | **CONEMO (Control Emotional)**: Culturally and linguistically adaptable smartphone app delivering 18 behavioral activation sessions across 6 weeks to promote daily life activities, motivation as well as to provide information and self-care messages. Provides: (1) psychoeducation about depression; (2) lists of potential activities to engage in; (3) motivational content; and (4) strategies to complete activities through text and videos. Includes interactive elements (notifications, SMS appointment reminders, dialogue pop-up reminders and feedback to complete app sessions; and option to request nurse support). Nurse dashboard facilitates nurse support for app orientation/training and motivational monitoring calls. | Participants found the CONEMO app usable, were satisfied with the app, and exhibited improvements in depressive symptom severity at the end of the intervention. Context-specific adaptations to protect nurse time are needed to implement the task-shifting approach. |
| Menezes, 2019 [69] | Feasibility, acceptability, and preliminary effectiveness  (n=66) |  |  |  |  | Participants were able to use the CONEMO app, were satisfied with the intervention, and demonstrated reduced symptoms of depression at the end of the intervention. CONEMO appears to be feasible to use for patients with diabetes and/or hypertension and comorbid depressive symptoms within 2 LMIC settings (São Paulo, Brazil, and Lima, Peru) with different languages. |
| Huberty, 2019 [88] | Feasibility  (n=128) | Depression | Cancer | Mobile device | **Calm & 10% Happier:** Meditation apps for the general population available on Android and iOS. The 10% Happier app’s introduction to meditation provides information for new meditators. The app includes short courses and daily individual guided meditations (~10-12 mins in length) of different foci (e.g., grief, gratitude, etc.) in the meditation library. The Calm app’s introduction to meditation includes information for new meditators and brief experiential practices. The app provides new and unique daily meditations (Daily Calm, ~10 to 12 min in length) of different foci (e.g., patience, loving kindness, etc.) which can be selected from the meditation library. Additional features include the Breathe Bubble, Sleep Stories, Calm Body, Calm Music, Calm Masterclass, and Background Scenes. | When comparing consumer-based smartphone meditation apps for use with myeloproliferative neoplasm patients, the Calm app demonstrated greater feasibility in terms of demand, acceptability than the 10% Happier app. Both apps demonstrated small effects on anxiety, depression, sleep disturbance, and total symptom burden. Differences in feasibility may be attributed to the design and organization of the Calm app (e.g., soothing narrator’s voice). |
| Huberty, 2019 [98] | Feasibility  (n=48) |  |  |  |  | Naive or nearly naive meditators with myeloproliferative neoplasm perceived meditations delivered via mobile phones to be enjoyable and beneficial for their mental health, sleep, fatigue, and pain. Patients preferred the Calm app over the 10% Happier app as the former app was perceived as more appealing due to specific design features (social media integration, automatic progress tracking, user statistics, and soothing sounds and backgrounds). |
| Greer, 2019 [80] | Efficacy  (n=145) | Anxiety | Cancer | Mobile device | **CBT Mobile App Intervention:** Mobile app delivering six sessions (20-30 min each) intended to be completed over 10-12 weeks (in order). Sessions consist of skill-building videos on managing cancer-related anxiety and simulations of patient-therapist interactions with homework exercises (10-15 mins each) corresponding to each session. A seventh session is included to review material from the previous sessions. | Improvements in anxiety, mood, and quality of life were observed for both the tailored CBT app and health education groups; although, there were no statistically significant differences in outcomes between groups. Participants with higher anxiety at baseline demonstrated greater improvements in anxiety from the CBT app compared to health education. Adherence to the app was low (completion of expected # of sessions below 75%). |
| Mohammad, 2019 [52] | Effectiveness  (n=80) | Anxiety | Cancer | VR | **Immersive VR:** VR exposure session through a head mounted display and headphones to provide distraction. Participants could select from two scenarios on CD-ROM, “Ocean Rift” (deep sea diving) and “Happy Place” (sitting on beach) with the VR exposure session ending at the peak of painkiller efficacy. | A single session of immersive VR in addition to morphine significantly reduced self-reported anxiety and pain among patients with breast cancer compared to morphine alone, suggesting its promise as a distraction technique. |
| Arch, 2020 [55] | Acceptability, feasibility, and efficacy  (n=35) | Depression & anxiety | Cancer | Web-based | **M-ACT:** Hybrid ACT intervention with alternating in-person and online sessions. Consists of (1) manualized in-person group sessions (4 sessions, 2 hours each) co-led by social worker and psychologist; (2) online sessions (3 self-paced sessions, 45 min each) with interactive exercises to apply skills learned in group sessions and content adapted to individual’s values/goals; and (3) daily online check-ins (5-7 min). Intervention aims to promote coping through active acceptance of emotions, reducing the dominance of distressing thoughts, clarifying personal values, and committing to activities and decisions that align with personal values. | With 92% of participants who began M-ACT completing the intervention and reporting high satisfaction, M-ACT intervention was feasible and acceptable to adults with metastatic cancer. A quarter of participants did not start the intervention mostly due to health declines. No changes in pain interference were observed but significant improvements in anxiety, depression, advanced care planning, sense of meaning, and fear of dying at 2-months follow-up demonstrated preliminary support for the efficacy of M-ACT. |
| Igelström, 2020 [81] | Acceptability  (n=15) | Depression & anxiety | Cancer | Web-based | **iCAN-DO:** Web-based stepped care program consisting of two steps. Step 1: nurse-led interactive e support program consisting of a psychoeducation library with 16 modules, peer support, chat, forum, FAQs, and an “Ask an Expert” section. Step 1 was available to participants for 24 weeks. Step 2: psychologist guided 10-week iCBT and peer support. iCBT participants received an introductory module after which they could select any of the 15 iCBT modules to complete during the 10-week period based on their personal relevance. 2-4 weeks were required for each module, which included psychoeducation, exercises, and assignments. Content in both steps were delivered through texts, audiovisual presentations, slide shows, and video clips. iCAN-DO was based on Orem’s self-care deficit nursing theory and social cognitive theory. | High accessibility and multiple delivery modes via responsive design were valued aspects of iCAN-DO. Barriers posed by cancer and external life circumstances placed added demands on the user experience. Technical challenges (multistep log in, need for add-ons) reduced motivation and usage. Perceptions of the design of iCAN-DO varied based on participants’ internet activity, computer experience, and interactions in social media. |
| Hauffman, 2020 [78] | Acceptability  (n=15) |  |  |  |  | iCAN-DO was perceived as a safe and reliable complement to standard care for cancer that provided information and support. Participants saw the platform as being most valuable to them and their kin at time of diagnosis when informational needs were high. Further personalization of the platform based on diagnosis, age, sex, treatment, and symptoms was desired. Most participants were not interested in peer support beyond lurking. |
| Hauffman, 2020 [82] | Effectiveness  (n=245) |  |  |  |  | Compared to standard care, iCAN-DO significantly reduced depressive symptoms and the proportion of people with cancer who had depressive symptoms at 10 months. No significant effects on anxiety, posttraumatic stress, or HRQoL were observed. |
| Koehler, 2020 [72] | Secondary analysis  (n=710) | Depression | Heart Failure | Telehealth system | **Telemedical Intervention:** Usual care and daily use of a telemonitoring system prompting daily self-measurements of 3-lead electrocardiogram, blood pressure, and weight with devices connected by secure Bluetooth. Personal digital assistant transfers data to the telemedical center through an integrated cell phone module with data also accessible to patient’s PCP. Regular monthly contacts provided by a member of the telemedical centre or as requested/needed by the patient. Emergency call system ensures 24/7 medical support via physicians at the telemedical center. Outpatient visits for physical examination, blood sampling, depression and quality of life assessment scheduled every 3 months and every 6 months in the first and second year, respectively. | Significant differences in depression between the telemedical group and the usual care group for patients with pre-existing moderate symptoms of depression. Individuals without pre-existing depressive symptoms at baseline had no significant changes in depression, indicating no negative impact of telemedical care on depressive symptoms. All patients receiving telemedical care reported significantly better quality of life compared to the usual care group regardless of level of depressive symptoms. |
| Chun, 2020 [60] | Feasibility  (n=27) | Anxiety | Stroke | Multiple technologies (Web-based, email, and text) | **TASK-CBT:** Guided self-help telemedicine program consisting of 6 weekly phone sessions (35-40 min each) with a TASK therapist (CBT-trained and supervised stroke physician). Between phone sessions, participants received an online task to practice and one or more psychoeducational videos to view on the TASK-CBT website. | CBT delivered through telemedicine was feasible to deliver to address anxiety after strokes. Clinical trial workflow is feasible to implement remotely. |
| Puzia, 2020 [95] | Secondary analysis  (n=80) | Depression & anxiety | Cancer | Mobile device | **Calm:** Mindfulness smartphone app with meditations based on MBSR and CBT techniques, encouraging users to practice non-judgemental present-moment awareness and to develop awareness of thoughts, emotions, and physiological responses to create more balanced thoughts. | The Calm mindfulness meditation app was effective in reducing depression and anxiety among patients with cancer, with those with the poorest global mental health at baseline experiencing the largest reductions. No significant differences in the changes in sleep disturbance were observed between the intervention and control groups. |
| Ye, 2020 [54] | Effectiveness  (n=154) | Depression & anxiety | Cancer | Mobile device | **Unnamed:** Informational video (2 mins) accessed through a QR code to reduce anxiety, depression, and pain through patient education on correct use of fentanyl transdermal patches. Video included cartoon images of the hospital, inpatient area, and a physician. Physician in the video explains areas to attend to before, during, and after using fentanyl transdermal patches (e.g., appropriate patch sites, skin preparation procedures, etc.). | Video education yielded statistically significant differences in state anxiety but not depression scores compared to conventional education for cancer patients receiving transdermal fentanyl patches for cancer pain. |
| Ali, 2020 [73] | Effectiveness  (n=404) | Depression | Diabetes (T2) | EHR | **Collaborative Care Intervention:** Included components at the patient- (self-care), clinician- (measurement-based care), and system-levels (monitoring patient panels, outreach to patients with poorly controlled health). To supplement usual care by diabetes physicians, each site had a team of non-physician care coordinators with backgrounds in allied health fields with no prior mental health training. Teams on each site received training in the care model, workflows, and use of decision support tool prior to study start. Care coordinators received training in behavioural activation, suicide risk management, motivational interviewing, and skills to support self-management, and had monthly coaching calls with study psychiatrist, psychologist, and nurse. To support patient self-care, care coordinators regularly contacted patients by phone/in-person every 2-4 weeks to monitor depressive symptoms (via PHQ-9), review blood glucose and/or blood pressure logs, and counsel patients towards their individual goals. Decision support tool integrated within EHR and clinic workflows to support diabetes physicians in clinical decision making and initiating of evidence-based therapies for diabetes and depression. Tool integrates patient characteristics, depression scores, lab results to deliver evidence-based clinical prompts using a built-in algorithm based on treatment guidelines and requires physicians to enter justifications for deviating from prompts in clinical decisions. At clinic level, clinics held caseload review meetings (every 2-4 weeks) with care coordinators and consulting specialists to review EHR data and make consensus recommendations for patients with poorly controlled depression/ cardiometabolic health. | A 12-month multicomponent collaborative care intervention yielded statistically significant improvements in depressive symptoms and cardiometabolic indices at 24 months for patients with diabetes and depression compared to usual care. During the active intervention phase (baseline-12 months), improvements were significantly larger with the between-group differences diminishing during the 12 months post-intervention phase. |
| Cohen, 2020 [96] | Efficacy  (n=30) | Depression | Diabetes (T1 or T2) | Telehealth system | **Health Buddy®:** Electronic device with monitor and modules enabling communication between patients and health care providers. Each day, the system provides a personalized greeting, asks about adherence to medication/self-care behaviours, and prompts the patient to take measurements  (vital signs, symptoms, and behaviours) using blood glucose monitors, scales, and blood pressure monitors. Educational or trivia questions about diabetes are presented before results are submitted but not reviewed by the care team. Data is sent to the nurse or pharmacist daily who can view values on a secure website and discuss care with the patient via phone. Patients also receive a two-part pharmacist-led telehealth visit. Part 1: Usual care visit (~45 mins) involving medication reconciliation and education on operating the Health Buddy® system and equipment via return demonstration. Part 2: Patient asked to bring all medications used (prescribed, non-prescribed, and alternative) for the telehealth pharmacist to answer questions, discuss administration and side effects, educate on anti-depressant and diabetes medications; and check dosages, omissions, and indications. Pharmacist also provided self-management education on blood glucose and weight assessment. Positive reinforcement and out-of-range results discussed via phone calls. Patient’s PCP notified of medication changes. | Compared to nurse-led telehealth, pharmacist-led telehealth resulted in no statistically significant improvements in overall depression scores, although improvements in adherence to cardiovascular, antidepressant, and overall medications was observed at 6-months follow up. |
| Kearney, 2021 [49] | Implementation & effectiveness  (n=1328 planned) | Depression & anxiety | Multimorbidity (At least 1 chronic physical condition & 1 chronic physical or mental health condition) | Mobile device | **Digital (High-Tech):** Digital care platform enabling (1) ~20-25 remote care interactions across a minimum of 4 months; (2) monthly video visits with a care manager; and (3) condition-specific check-ins via text messages (brief 1-4 question assessments at a daily, weekly or biweekly frequency depending on the condition). Care manager is alerted to check in with participants with parameters beyond the normal range. | Rapid evaluation and quality improvement processes, redesigned workflows, and tailored approaches are needed to successfully implement in-person and technology-based care strategies for multiple chronic conditions. |

ACT: Acceptance and Commitment Therapy

CBT: Cognitively Behavioural Therapy

EHR: Electronic Health Records

FAQ: Frequently Asked Question

GAD-2: 2-item Generalized Anxiety Disorder scale

GPS: Global Positioning System

HbA1c: Haemoglobin A1c

HIPAA: Health Insurance Portability and Accountability Act

iCBT: Internet-based Cognitively Behavioural Therapy

IPT: Interpersonal Therapy

MBSR: Mindfulness-based Stress Reduction

PCP: Primary Care Provider

PHQ-2: 2-item Patient Health Questionnaire

PHQ-8: 8-item Patient Health Questionnaire

QR: Quick Response

SDS: Sheehan Disability Scale

VR: Virtual Reality
